# Reframing health inequality? The rise, rise and fall of three competing policy frames

**DOI:** 10.1093/pubmed/fdaf141

**Published:** 2025-11-03

**Authors:** Ally Brown

**Affiliations:** School of Social Work and Social Policy, University of Strathclyde, 141 St James Road, Glasgow G4 0LT, UK

**Keywords:** inequality, policy, health inequality, framing

## Abstract

**Background:**

Since the end of New Labour’s health inequality strategy, health inequalities in the UK have been widening. A recent critique suggested that New Labour policy actors were misdirected towards less effective solutions by the ‘international consensus’ understanding of health inequalities, which counterproductively medicalizes social inequality. This research explored whether social, economic and health policy actors at devolved levels shared this ‘international consensus’ policy frame of health inequality.

**Methods:**

The Scottish Government and Greater Manchester Combined Authority were chosen as case study polities. Thirty-four policymakers were interviewed, and a frame analysis was conducted of thirty social, economic and health policy strategy texts, published between 2017 and 2022.

**Results:**

The ‘international consensus’ policy frame was supported in these contexts by strong moral language, highlighting the social injustice of systematically distributed illness and death. However, political support was building behind health inequality framed in relation to ‘illness-related economic inactivity’ and ‘healthcare for disadvantaged groups’. These two alternative frames using the same term directed policy solutions towards individual-level reactive healthcare, rather than population-level preventive public policy.

**Conclusions:**

‘Health inequality’ is understood in three competing ways in these policy settings. Alternative terms, such as ‘social inequalities in health’ and ‘healthcare inequality’, are preferable to minimize ambiguities.

## Introduction

Following the apparent failure of New Labour’s health inequality strategy for England, researchers faced a dispiriting question with a seemingly negative answer: “Can we reduce health inequalities?”.^1^ For “*the vast majority*” of the health inequality research and policy community interviewed by Smith and Garthwaite,^2^ the strategy had produced disappointing outcomes. Yet, with the benefit of better data, a systematic review more than a decade after the end of the strategy seemed to answer differently: Holdroyd et al.’s 2022 analysis^3^ showed that strategic targets aimed at inequalities in life expectancy and infant mortality had in fact been met, at least partly.

In the meantime, a succession of Conservative-led governments had taken power, with less interest in reducing health inequalities. Their economic austerity agenda, followed by the Covid-19 pandemic and a rapid rise in inflation, all contributed to widening inequalities.^4–6^ In 2024, a new Labour government won a landslide electoral victory, with a manifesto commitment to reducing healthy life expectancy gaps in England. At the time of writing, it is not clear how the national government intends to achieve this reduction.

An important recent critique, first articulated in this journal, develops an alternative perspective of the health inequalities agenda: that the ‘international consensus’ understanding of health inequalities counterproductively medicalizes social inequality. According to Lynch’s analysis,^7^^,^^8^ New Labour’s attention to ‘health inequality’ signalled to voters a concern with inequality, but implied solutions via the notoriously troublesome tasks of joined-up government, and in healthcare, rather than by economic redistribution. Therefore, by viewing ‘health inequalities’ as a political term, it may be seen as a ‘*dangerous frame shift*’ that misdirects minds away from effective policy solutions.

Although policy responsibility for health inequalities is routinely attributed to health policy teams, reducing inequalities in the social determinants of health requires action from social and economic policy teams. Therefore, this research builds on Lynch’s work to explore whether the ‘international consensus’ understanding of health inequalities among researchers is shared by social, economic and health policy actors in two sub-state devolved polities in the UK.

## Methods

The Scottish Government (SG) and Greater Manchester Combined Authority (GMCA) were chosen as case studies due to their declared interest in reducing health and social inequalities,^9^^,^^10^ and their status as devolved polities. Documents and actors from closely affiliated agencies, such as Public Health Scotland and Transport for Greater Manchester, were also considered for inclusion.

Then, NVivo was used to conduct a frame analysis of thirty social, economic and health policy texts published in these two settings between 2017 and 2022. Included texts are presented in Tables 1 and 2, below. Following Lynch and others, this involved categorising data representing each of the five core elements of a policy frame within each document—‘causal stories’, ‘problems’, ‘solutions’, ‘actors’, and ‘moral language’—relating to either health, or to inequalities.

**Table 1:** Greater Manchester texts analysed.

**Document**	**Policy Area**	**Year**
Our People, Our Place: The GM Strategy	Overall Strategy	2017
Children & Young People’s Health and Wellbeing Framework	Children & Young People’s Health	2018
Taking Charge: the Next 5 Years	Health	2019
Local Industrial Strategy	Economic Development	2019
Housing Strategy	Housing	2019
Children and Young People’s Plan	Children & Young People	2019
GM Model for Public Services	Public Services	2019
Transforming the Health of our Population	Health	2019
Transport Strategy 2040	Transport	2021
Local Skills Report & Labour Market Plan	Skills & Employment	2021
AGMA Places for Everyone	Spatial Planning	2021
Good Lives for All: GM Strategy 2021–2031	Overall Strategy	2022

**Table 2:** Scottish texts analysed.

**Document**	**Policy Area**	**Year**
Mental Health Strategy 2017–2027	Mental Health	2017
Every Child, Every Chance: Tackling Child Poverty Delivery Plan 2018–2022	Equalities/Children	2018
No One Left Behind: Next Steps for Employability Support	Skills/Employment	2018
Public Health Priorities for Scotland	Public Health	2018
National Transport Strategy	Transport	2020
Economic Recovery Implementation Plan: SG Response to Advisory Group on Economic Recovery	Economic Development	2020
Public Health Scotland’s Strategic Plan 2020–2023	Public Health	2020
Updated Climate Change Plan 2018–2032	Climate	2020
A Fairer, Greener Scotland: Programme for Government 2021–22 (Non-Health Chapters)	Social and Economic Policy overview	2021
A Fairer, Greener Scotland: Programme for Government 2021–22 (Health Chapter)	Health Policy overview	2021
Scottish Government Vision For Trade	Economic Development	2021
Fair Work Action Plan	Employment/Equalities	2021
Social enterprise Action Plan	Skills/Employment	2021
Public Health Scotland delivery plan 2021–24	Public Health	2021
Housing to 2040	Housing	2021
Covid Recovery Strategy: for a fairer future	Overall Strategy	2021
Scotland 2045: Our Fourth National Planning Framework Draft	Spatial Planning	2021
Delivering Economic Prosperity: National Strategy for Economic Transformation (NSET)	Economic Development	2022

Further, semi-structured interviews were conducted with thirty-four policy actors working in these settings. All interviews were conducted online using Microsoft Teams. Participants were asked a range of questions relating to health, inequalities, and to health inequalities, for between 45 and 88 minutes. Transcripts were anonymized and then coded both deductively and inductively.

This research was approved by the University of Strathclyde’s Research Ethics Committee.

## Results

In addition to the ‘international consensus’ health inequality policy frame identified by Lynch, this research identifies two further policy frames used to conceptualize health inequalities within these policy settings. These two additional policy frames compete with the ‘international consensus’ frame in the sense that they use the same term—health inequality—to promote a different set of policy solutions. These three frames compete—with each other and with other claims about social problems—for policymaker attention and resource.^11^^,^^12^

### The international (research) consensus frame: health inequality as social injustice

The ‘international consensus’ frame described by Lynch (Table A, below) reflects a research consensus developed over several decades, and is strongly associated with the work of Professor Sir Michael Marmot. Social injustice is at the heart of this version of health inequality: it represents the unprevented social distribution of ill-health suffering to millions of people.

**Table A:** International (research) consensus policy frame (adapted from Lynch^8^).

**Element of policy frame**	**‘International Consensus’ Policy Frame**
Causal Story	Social inequality is the key driver of health inequality
Problem	Health inequalities are avoidable and unfair differences in health status between socioeconomic groups
Solutions	Joined-up government policy is required to reduce inequalities in the social determinants of health
Actors	Governments—particularly national governments—are responsible for reducing social, economic and health inequality
Moral responsibility	Economic and social forces—including un-regulated capitalism—that generate social inequality are to blame; and governments for failing to mitigate these forces

Understood this way, the widespread social injustice of ‘health inequalities’ carries a powerful ‘moral sting’, which is evident in much of the moral language coded in the policy texts. Often the strongest moral language in each policy text analysed related to health inequality:

“*Deeply embedded health inequalities, often between communities little more than a stone’s throw apart, have blighted individual lives and acted as a drag on our economy*” (Our People, Our Place, GM, 2017, p60)

“*The impact [of Covid] has been unequal and unfair, starkly highlighting and deepening the inequalities we know have existed for many years and which we were beginning to change*” (Places for Everyone, GM, 2021, p8)

“*None of this is acceptable. Despite tremendous progress in life expectancy it is not acceptable that our health is poorer than other parts of Europe, and it is not acceptable that people in Scotland are not able to thrive.*” (Public Health Priorities, SG, 2018, p6)

The moral perspective conveyed by the above policy text excerpts was also expressed passionately by a senior health policymaker in Scotland as follows:

*“I fundamentally think that Scotland’s challenge is on health inequalities rather than improving population health or in system sustainability [...] I might mention a moral requirement which is maybe somewhere where you’ll get the passion, which is fundamental, if you ask me, what is my basic thought and what gets me up in the morning […] I have no particular interest in just the question of raising the tide of health in the population […] there are people systematically dying in their 40s and 50s and that’s a scandal”* [SCOP07H]

But this policymaker, and others in Scotland, sensed that this version of ‘health inequality’ was struggling for political attention:

“*I do think health inequalities as a framing generally is tired and risks having run out of road.”* [SCOP07H]

“*But* health inequalities as an inequality *if you like is, I would say, a little bit less obvious. I’d say it flies a wee bit more under the radar.”* [SCOP04O, emphasis added]

“*We are acutely aware that people are not talking in the policy space around falling life expectancy, widening healthy life expectancy [gaps] or the [social] gradient [in health]*” [SCOP14H]

Further, this understanding of health inequality does not reflect a policy consensus: two other health inequality policy frames were identified and are described below. The key difference between this frame and the two others is that this frame attributes moral responsibility for the origin of illness to governments, which creates a political story or ‘narrative’^13^ about systematic variations in population health. On the other hand, the others attribute responsibility only to services to react to illness, without taking interest in its origins, which portrays those variations as apolitical matters of service provision.

### The illness-related economic inactivity frame: health inequality as population-scale illness

In contrast to health policy participants, social and economic policy participants commonly conceptualized health as illness, and further used ‘health inequality’ to mean widespread illness. Particularly for economic policy participants, this meant ‘health inequality’ was conceptualized as a cause of economic problems, as illness was an impediment to work (see Table B, above). For example, in this quote a senior Scottish policymaker involved in the child poverty agenda recounts reframing ‘health inequalities’ from a problem to a cause of problems in an interaction with a health policy colleague:

**Table B:** Illness-related economic inactivity policy frame.

**Element of policy frame**	**Illness-related Economic Inactivity Policy Frame**
Causal Story	Widespread illness is weakening the labour market, leading to unfilled job vacancies and skills shortages.
Problem	Economic inactivity is weakening economic productivity.
Solutions	Quicker access to more effective healthcare reduces illness and allows individuals to re-enter the labour market.
Actors	Healthcare providers must work with state employability services to better target healthcare solutions for individuals.
Moral responsibility	There is no moral responsibilisation for illness, but the state and healthcare actors have responsibility to manage the treatment of illness.

“She’ll say to me, ‘yes I’m really interested to know about how the work you’re taking forward in the child poverty sphere will help me with health inequalities’, and I’m like ‘well actually no, it’s what you’re doing in terms of tackling health inequalities will help the child poverty work’. Because obviously if we’ve got a healthier population they’re more likely to be able to take up work” [SCOP03O]

Unlike the ‘international consensus’ frame, this frame is not concerned with socioeconomic groups, avoidable differences in health, or the role of social inequality as a key driver. Rather, here the problem is economic inactivity, and the causal story is the disabling impact of ill-health on employment. In the policy texts analysed with features of this frame, and from interviews with relevant policy participants, there is no overt moral responsibilisation, no causal explanation for illness, and the policy solution is medical.

This illness-related economic inactivity policy frame meant that health inequality was described by economic policy participants as a much higher political priority than it had been previously. Several participants in Greater Manchester highlighted an economic report from 2019, the Greater Manchester Independent Prosperity Review,^14^ as being key for highlighting the impact of poor health on the economic performance of the city.

“*I think health inequalities have definitely become a much bigger part of all the debate in Greater Manchester across policy areas*.” [GMP08O]

Scottish economic policy participants also described health inequality—understood in this way—as a much higher political priority. In the quote below, where it is described as ‘*a huge priority*’, a senior employability policy participant explicitly uses the expiration of a healthcare program to reframe an intended focus on social inequalities as a focus on ‘health inequalities’:

Q: How much of a priority is health inequalities in your team? A: “*It's a huge priority. So we have, within my area I have three different strands of work. One of them is ‘tackling inequalities’ but that really is health inequalities. Part of the reason for the focus on that now is […] the key health offer for employability […] ends in March 2024.”* [SCOP21O]

Additional interest by economic policy teams in health may provide opportunities for collaborative policy development for win-win outcomes.^15^ On the other hand, there are several plausible concerns about an illness-related economic inactivity frame. For example, many disabling illnesses cannot be quickly or easily improved, and a reactive, individual-level approach cannot sustainably manage demand. Further, an employment focus arguably narrows the policy understanding of health to whatever functions are required for employment, and no more. Third, such a policy frame prioritizes the potential labour force, to the potential detriment of policy attention to other important population groups, such as children, people with caring responsibilities, people with severe disabilities, prisoners, asylum seekers, and retirees.

### The healthcare for disadvantaged groups frame: health inequality as healthcare inequality

In this research, ‘health’ was also widely used in social and economic policy settings as shorthand for ‘health policy’, which is dominated by healthcare. Therefore, ‘health inequality’ was commonly used as if to mean ‘healthcare inequalities’, particularly in Scotland (see Table C, above).

**Table C:** Healthcare for disadvantaged groups policy frame.

**Element of policy frame**	**Healthcare for Disadvantaged Groups Policy Frame**
Causal Story	Disadvantaged people have less access to adequate healthcare
Problem	Health inequality is (at least partly) caused by inadequate healthcare for disadvantaged groups
Solutions	Improving access to and treatment from healthcare providers for disadvantaged groups
Actors	Healthcare providers and the government should work to better target healthcare provision and improve outcomes
Moral responsibility	There is no moral responsibilisation for illness, but the state and healthcare actors have responsibility to ensure the treatment of illness is available to all.

Only three of the 11 health policy participants mentioned ‘healthcare inequality’ without prompting, and each mention was specifically to distinguish between healthcare and social inequalities, as in this comment by a senior Scottish policymaker:

*“So I’m not obsessed by healthcare inequality, and by health inequality I mean something that I don’t particularly differentiate from social inequality either.”* [SGP07H]

The features of this frame were highlighted by health policy actors in GM and Scotland, who described NHS England-driven work on healthcare inequalities substituting for policy attention to social inequalities in health. In GM, one participant described “*the NHSE health inequality plan, which is very much around reducing variation in care... focusing on clinical priorities and deprived populations and making sure we’re addressing health inequalities through our healthcare*” [GMP12H]. In Scotland, attempts to broaden NHS England’s healthcare inequalities approach Core20PLUS5 to encompass wider social inequalities in health failed “*for various reasons, and the latest I’ve seen is we’ve gone back to that healthcare inequalities stance*” [SCOP05H].

Remarkably, not one of the 23 social or economic policy interviewees ever mentioned ‘healthcare inequality’. This may simply reflect the very common conflation of ‘health’ with ‘healthcare’; it may be habitual, due to the common use of the term ‘health inequality’; or it may seek to borrow some of the extra ‘moral sting’ of the ‘international consensus’ policy frame, described in 3a, above.

‘Health inequality’ was confused with ‘healthcare inequality’ in at least two Scottish Government policy teams. Firstly, the Health Inequalities Unit within DG Health included three teams, one of which was the Prisoner Healthcare team. Secondly, a new Racialised Health Inequalities team had been established, though its sole focus was healthcare inequalities. It took repeated questioning to ascertain that this team was not focused on racism as a social determinant of ill-health:

“Really the name should be Racialised Healthcare Inequalities, but it isn’t at the moment and they’re not minded to change it.”

This policy frame bears similarities to the illness-related economic inactivity policy frame, particularly if the unwell unemployed are conceptualized as a disadvantaged group. But the focus of the healthcare for disadvantaged groups policy frame is on improving healthcare provision for its own sake, rather than for the sake of achieving employment. Therefore, it does not prioritize those whose economic inactivity is primarily related to ill-health: this can be seen by the location of a Prisoner Healthcare team within the Health Inequalities Unit.

The particular prominence of this policy frame in Scotland may at least partially reflect the advocacy of the ‘Deep End’ group of GPs in deprived communities, who have admirably and effectively campaigned for policy action to address the inverse care law^16^ since 2009. Despite their focus on primary care, a recent major Deep End report mentions ‘health inequality’ eighty times, but not ‘healthcare inequality’ at all.^17^

In Scotland, this policy frame achieved support at the highest level when new First Minister Humza Yousaf announced an investment of one million pounds in primary care services in deprived areas, under the headline ‘Tackling health inequalities’.^18^ Describing policy attention to healthcare inequality as ‘tackling health inequalities’ misleadingly implies that action is being taken on inequalities in social determinants of health. Therefore, this policy frame may undermine political will to act on social inequalities, while reinforcing medicalized understandings of health.

In contrast, following the conclusion of this research, the Scottish Government promised to develop a new ‘Healthcare Inequalities Action Plan’,^19^ which may indicate a new effort towards conceptual clarity.

## Discussion

### Main findings of this study

This study finds policy actors using ‘health inequality’ in three different ways. First, the ‘international consensus’ understanding of researchers is largely reflected by health policy actors, where the additional ‘moral sting’ of social injustice is evident in strong moral language. But this policy frame was struggling for attention in both contexts, while two competing versions of ‘health inequality’ gained support. In economic policy teams, ‘health inequality’ was used to mean widespread illness impeding employment. Here, the problem to be solved is unemployment; the origins or patterning of ill-health is not known; and the solutions are medical. Third, ‘health inequality’ was used in reference to healthcare provision for disadvantaged groups. While this version of health inequality acknowledges social disadvantage, it also situations solutions within healthcare services, rather than within the social conditions in which people live.

### What is already known on this topic

The terms ‘health inequalities’, ‘health disparities’, ‘health variations’, and similar have been debated at length.^20^^–^^22^ To a large extent, disagreements over meaning originated geographically, with UK-based researchers preferring the term ‘inequalities’ since its use in the official title of the highly influential Black Report,^23^ while US-based researchers used ‘disparities’ to reflect observed gaps in healthcare provision between white and minority ethnic groups.^24^

As described in the introduction, Lynch^7–8^ explored the political uses and framing effects in policy settings of the term ‘health inequalities’. She identified an ‘international consensus’ frame developed by researchers, and concluded that in policy settings it counter-productively directed solutions towards healthcare, and complex joined-up government arrangements.

### What this study adds

This study built on Lynch’s work to explore how the term ‘health inequalities’ is used in two distinct policy settings, finding two additional ‘versions’ of the term, preferred by different groups. This finding suggests that policy communications about ‘health inequalities’ might not mean what researchers, other policy actors, or the public take them to mean. These three policy frames are based on different causal stories, focus on different problems, apply moral responsibility differently, and imply different solutions from different actors. Importantly, these three frames compete—with each other and with other claims about social problems—for policymaker attention and resource.

In both policy settings, ‘health inequality’ was described as being newly prioritized. However, this prioritisation was for illness-related economic inactivity, and healthcare for disadvantaged groups. Both the prioritized conceptualisations implied policy solutions in reactive individual-level healthcare, rather than preventive population-level social policy.

Despite the strong moral language used to describe the social injustice of systematically distributed illness and death, this version of ‘health inequality’ was not politically prioritized: it was ‘tired’, ‘under the radar’, and almost ‘out of road’. Therefore, research and policy actors opposed to this social injustice should consider using alternative, less ambiguous terms, such as ‘social inequalities in health’, ‘widespread ill-health’, and ‘healthcare inequality’, to more precisely differentiate between these policy frames, and to better communicate their aims and ideas. Further, actors must consider alternative frames or narratives which might better mobilize political support for systematic solutions to preventable ill-health suffering, while also contesting those which reinforce individual-level, reactive solutions.

### Limitations of this study

This research is limited to two settings: GMCA and the Scottish Government. Policymakers were unavailable for participation in some important policy domains, including employment, spatial planning and climate policy in Greater Manchester, and devolution arrangements in Scotland. There may have been an element of self-selection bias in these non-responses; equally, participants may be particularly interested in health inequality. Therefore, further research identifying these policy frames—or others—in additional policy settings would be beneficial, particularly in international settings where policy frames may be shaped differently by linguistic or cultural variations.

## Data Availability

The data that support the findings of this study are available from the corresponding author upon reasonable request.

## Notes

**Ally Brown**, Research Associate
